# Many rice genes are differentially spliced between roots and shoots but cytokinin has minimal effect on splicing

**DOI:** 10.1002/pld3.136

**Published:** 2019-05-17

**Authors:** Nowlan H. Freese, April R. Estrada, Ivory C. Blakley, Jinjie Duan, Ann E. Loraine

**Affiliations:** ^1^ Department of Bioinformatics and Genomics University of North Carolina at Charlotte Charlotte North Carolina; ^2^ Department of Biomedicine Aarhus University Aarhus C Denmark

**Keywords:** alternative splicing, cytokinin, rice, RNA‐Seq, root, shoot

## Abstract

Alternatively spliced genes produce multiple spliced isoforms, called transcript variants. In differential alternative splicing, transcript variant abundance differs across sample types. Differential alternative splicing is common in animal systems and influences cellular development in many processes, but its extent and significance is not as well known in plants. To investigate differential alternative splicing in plants, we examined RNA‐Seq data from rice seedlings. The data included three biological replicates per sample type, approximately 30 million sequence alignments per replicate, and four sample types: roots and shoots treated with exogenous cytokinin delivered hydroponically or a mock treatment. Cytokinin treatment triggered expression changes in thousands of genes but had negligible effect on splicing patterns. However, many genes were differentially spliced between mock‐treated roots and shoots, indicating that our methods were sufficiently sensitive to detect differential splicing between data sets. Quantitative fragment analysis of reverse transcriptase‐PCR products made from newly prepared rice samples confirmed 9 of 10 differential splicing events between rice roots and shoots. Differential alternative splicing typically changed the relative abundance of splice variants that co‐occurred in a data set. Analysis of a similar (but less deeply sequenced) RNA‐Seq data set from *Arabidopsis* showed the same pattern. In both the *Arabidopsis* and rice RNA‐Seq data sets, most genes annotated as alternatively spliced had small minor variant frequencies. Of splicing choices with abundant support for minor forms, most alternative splicing events were located within the protein‐coding sequence and maintained the annotated reading frame. A tool for visualizing protein annotations in the context of genomic sequence (ProtAnnot) together with a genome browser (Integrated Genome Browser) were used to visualize and assess effects of differential splicing on gene function. In general, differentially spliced regions coincided with conserved protein domains, indicating that differential alternative splicing is likely to affect protein function between root and shoot tissue in rice.

## 1 INTRODUCTION

Alternative splicing of pre‐mRNA transcripts enables one gene to produce multiple transcript variants encoding different functions. Alternative splicing is an almost universal phenomenon in higher eukaryotes, occurring to varying degrees in every animal and plant genome examined to date (Kalsotra & Cooper, 2011; Reddy, Marquez, Kalyna, & Barta, 2013). In animals, differential expression of splice variants has been recruited as a regulatory mechanism in multiple processes, such as sex determination in invertebrates and neuronal differentiation in mammals (Barbosa‐Morais et al., 2012; Kalsotra & Cooper, 2011; Salz, 2011).

In plants, less is known about the functional significance and patterns of alternative splicing. However, several trends are apparent. Genes involved in circadian regulation are highly alternatively spliced, often producing multiple splice variants that fluctuate in concert with day/night cycling along with overall transcript abundance (Filichkin, Cumbie, et al., 2015). The serine and arginine‐rich (SR) family of RNA‐binding, splicing regulatory proteins is greatly expanded compared to mammals and includes many plant‐specific forms (Barbosa‐Morais, Carmo‐Fonseca, & Aparício, 2006; Filichkin, Priest, Megraw, & Mockler, 2015; Kalyna & Barta, 2004; Plass, Agirre, Reyes, Camara, & Eyras, 2008). SR transcripts themselves are also highly alternatively spliced in plants, with the relative abundance of these alternative transcripts varying according to environmental stresses and hormones (Filichkin, Priest, et al., 2015; Gulledge, Roberts, Vora, Patel, & Loraine, 2012; Keller et al., 2016; Mei, Boatwright, Feng, Schnable, & Barbazuk, 2017; Palusa, Ali, & Reddy, 2007).

A growing body of evidence indicates that cell and tissue specific regulation of alternative splicing occurs in plants, but its significance and extent is not well established (Li, Yamada, Han, Ohler, & Benfey, 2016; Sun et al., 2018; Vitulo et al., 2014). We previously found through analysis of RNA‐Seq data from *Arabidopsis* pollen that the relative abundance of splice variants was similar between leaves and pollen, despite the differences between the two tissues (Loraine, McCormick, Estrada, Patel, & Qin, 2013). However, this latter analysis was limited by having just one biological replicate for pollen and only two biological replicates for leaves. A more comprehensive analysis of multiple *Arabidopsis* data sets found a high incidence of isoform switching, in which the identity of the most prevalent variant differs between sample types (Vaneechoutte, Estrada, Lin, Loraine, & Vandepoele, 2017). This splicing diversity may have arisen in part from the heterogeneity of the data sets used, which were produced using rapidly changing (and improving) sequencing technologies at different times by different groups.

In this study, we used a well‐replicated RNA‐Seq data set from rice to re‐examine prevalence of alternative splicing between tissues and hormone (cytokinin) treatment. This data set was previously generated to investigate cytokinin regulation of gene expression in roots and shoots from 10‐day‐old rice seedlings (Raines et al., 2016). A parallel study produced an analogous data set from *Arabidopsis* for comparison, but was less deeply sequenced (Zubo et al., 2017). Both the rice and *Arabidopsis* RNA‐Seq data sets included three biological replicates per sample type and four sample types—roots and shoots treated with exogenous cytokinin or a mock, vehicle‐only treatment. In both data sets, the treatment triggered differential expression of thousands of genes, with roots affected to a greater degree than shoots.

For most alternatively spliced genes, regardless of whether or not they were differentially spliced, the relative abundance of splicing forms was highly skewed, with most alternatively spliced genes producing one major form. Nonetheless, there were many alternatively spliced genes where minor forms were abundant and therefore seemed likely to affect gene function. We found that the relative abundance of transcript variants for most alternatively spliced genes was remarkably stable, with very few differentially spliced genes between cytokinin treated and control samples. By contrast, many more genes were differentially spliced between roots and shoot, and most differential splicing occurred within the protein‐coding sequence. Moreover, nearly every differential splicing event detected changed the relative abundance of splice variants that co‐occurred in the same sample. These results suggest differential alternative splicing likely contributes to gene function diversification between roots and shoots by moderating the relative abundance of co‐expressed splice variants, but plays little role in cytokinin signaling.

## 2 MATERIALS AND METHODS

### 2.1 RNA‐Seq data

RNA‐Seq data from rice and *Arabidopsis* shoot and root tissue were from our previous studies on the effect of cytokinin on gene expression (Raines et al., 2016; Zubo et al., 2017). Rice sequence data are available from the Sequence Read Archive under accession SRP049054. Aligned, processed data are available from October 2011 rice genome assembly IgbQuickload directories at http://lorainelab-quickload.scidas.org/rnaseq/. *Arabidopsis* sequence data are available from the Sequence Read Archive under accession SRP059384. Aligned, processed data are available from the TAIR10 (June 2009) *Arabidopsis* genome assembly IgbQuickload directories at http://lorainelab-quickload.scidas.org/rnaseq/.

### 2.2 Data processing

Rice sequences were aligned onto the *Oryza sativa japonica* genome assembly Os‐Nipponbare‐Reference‐IRGSP‐1.0 (Kawahara et al., 2013) and *Arabidopsis* sequences were aligned onto the TAIR10 June 2009 release of the *Arabidopsis* genome (Lamesch et al., 2012) using TopHat (Kim et al., 2013) and BowTie2 (Langmead & Salzberg, 2012) with maximum intron size set to 5,000 bases. A command‐line, Java program called “FindJunctions” was used to identify exon‐exon junctions from gapped read alignments in the RNA‐Seq data. FindJunctions produces BED format files containing junction features, and the score field of the BED file lists the number of read alignments that supported the junction. Only reads that aligned to a unique location in the genome were considered. Source code and compiled versions of FindJunctions are available from https://bitbucket.org/lorainelab/findjunctions.

### 2.3 Identification of alternative splicing events and differential splicing

To date, there have been two major releases of *O. sativa japonica* gene models: the MSU7 gene set (Kawahara et al., 2013) and the RAP‐Db gene set (Sakai et al., 2013). The two gene model sets contain mostly the same data, but the MSU7 gene models appear to be the most heavily used and annotated with Gene Ontology terms. For simplicity, and to take advantage of available functional annotations, we used the MSU7 annotations here. For analysis of *Arabidopsis* data, we used TAIR10 (Lamesch et al., 2012) and Araport11 (Cheng et al., 2017) gene models.

**Figure 1:**
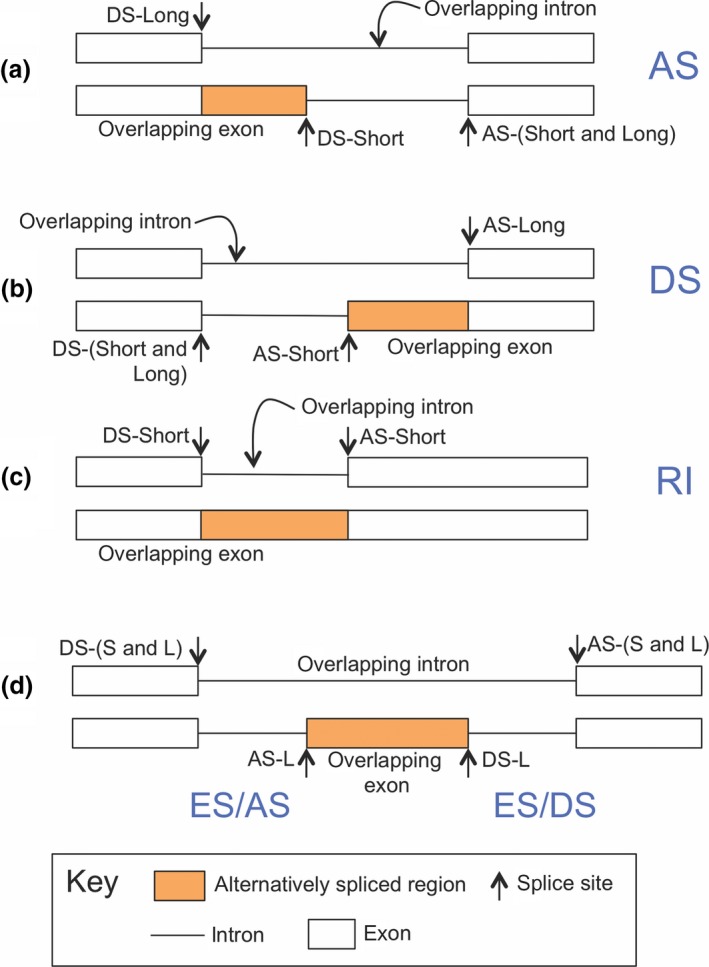
Alternative splicing annotation. The overlap between an intron in one gene model and an exon in another gene model defines an alternatively spliced region. Arrows indicate splice sites, named AS for acceptor site and DS for donor site. Use of sites named AS‐L or DS‐L causes inclusion of the differentially spliced region, generating the longer (L) form. Similarly, DS‐S and AS‐S refer to sites that exclude the differentially spliced region and generate the shorter (S) form. (a) Alternative donor sites, in which the U2 snRNP complex forms at alternative locations on the 5′ end of introns. (b) Alternative acceptor sites, in which the U1 snRNP complex forms at alternative sites near the 3′ end of alternatively spliced introns. (c) Alternatively spliced intron, in which a donor/acceptor site pairing can either be used or not used, forming a retained intron (RI). (d) Alternatively spliced, skipped exon. In exon skipping, alternative splicing involves four sites, indicated by DS‐S/L, AS‐L, DS‐L, and SD‐S/L. Exon inclusion requires assembly of two spliceosome complexes linking DS‐S/L with AS‐L and DS‐L with AS‐S/L, while exon skipping requires linking DS‐S/L and AS‐S/L only

Annotated alternative splicing events and the number of reads supporting each alternative were identified using the exon‐intron overlap method introduced in English, Patel, and Loraine (2010) and further developed here for use with RNA‐Seq data. Exons and introns from pairs of gene models from the same locus were compared to identify alternatively spliced regions. Regions where an intron in one model overlapped an exon in another model on the same strand were identified and used to define mutually exclusive splicing choices (Figure 1). Gene models that included an alternatively spliced region were designated the “L” form (for “Long”) with respect to the splicing choice. Likewise, models that lacked an alternatively spliced region were designated “S” (for “Short”). The major form was defined as the transcript variant with the highest expression when comparing pairs of gene models. Thus, the minor form had lower expression in comparison to the major form.

Alternatively spliced regions were labeled according to the type of alternative splicing, as follows. Regions flanked by alternative donor sites were designated “DS” for alternative donor site. Regions flanked by alternative acceptor sites were labeled “AS” for alternative acceptor site. AS and DS events that coincided with exon skipping were labeled “AS/ES” and “DS/ES”. Alternatively spliced regions arising from introns that the spliceosome sometimes failed to excise were designated “RI” for retained intron.

For each alternatively spliced region representing two mutually exclusive splicing choices, RNA‐Seq read alignments that unambiguously supported one or the other splicing choice were counted. For AS and DS events, only gapped reads that aligned across intron junctions were counted. For RI events, gapped reads that aligned across the retained intron were counted as support for the intron‐removed (S) form, and un‐gapped reads that overlapped at least 20 bases within the intron were counted as support for the intron‐retained (L) form.

For each alternatively spliced region in each biological replicate, the number of reads supporting L or S, were used to calculate percent‐spliced‐in (PSI) as 100*L/(S + L), where L was the number of reads supporting only the L form and S + L the number of reads that supported S or L but not both. This is the same as the splicing index described in Katz, Wang, Airoldi, and Burge (2010). To identify differentially spliced regions, a two‐sided *t* test was used to compare PSI between sample types. Because PSI variance was large for events with small M (very few informative reads), only alternatively spliced regions where M was 10 or more in at least three replicate libraries were tested. A false discovery rate (FDR) was calculated for each test using the method of Benjamini and Hochberg (Benjamini & Hochberg, 1995), as implemented in the R programming language “p.adjust” method. Alternative splicing events with FDR ≤0.1 were considered differentially alternatively spliced.

Software used to identify and quantify alternative events is available from https://bitbucket.org/lorainelab/altspliceanalysis. Data analysis code used to analyze RNA‐Seq data is available from https://bitbucket.org/lorainelab/ricealtsplice. Data analysis code is implemented as R Markdown files designed to be run in the RStudio development environment. Readers interested in experimenting with different analysis parameters can clone the repository, modify the code, and re‐run analyses as desired. RNA‐Seq alignments, coverage graphs, and junctions data are available for visualization in Integrated Genome Browser (Freese, Norris, & Loraine, 2016).

### 2.4 RT‐PCR and capillary gel electrophoresis analysis of alternative splicing

Differential alternative splicing detected by analysis of RNA‐Seq was re‐tested using the reverse transcriptase, PCR‐based fragment analysis method described in (Stamm, Smith, & Lührmann, 2012). Differentially spliced regions identified computationally were PCR‐amplified using fluorescently labeled primers and quantified using capillary gel electrophoresis. One benefit of the method is that the results are expressed as relative abundances of splice variants within a sample, thus eliminating the need to normalize using reference genes as in traditional qRT‐PCR experiments aimed at measuring overall gene expression.

For splicing validation, new rice seedlings equivalent to the mock‐treated (control) samples from the RNA‐Seq experiment were grown and harvested. Seedlings were grown hydroponically in pots containing either liquid media only or calcined clay granules watered with liquid media as recommended in (Eddy, Hahn, & Moulton, 2012). After 12 days, plants were removed from the pots and roots and shoots were collected separately. Roots and shoots from the same pot were combined to form paired biological replicates. Samples were flash frozen in nitrogen and stored at −80°C prior to RNA extraction.

RNA was extracted using the RNeasy Plant Mini Kit from Qiagen following the manufacturer's instructions, including the optional DNase I digestion to eliminate DNA contamination. First strand cDNA was synthesized using oligo dT primers and 1 μg of total RNA per 20‐μl reaction. PCR amplification of cDNA was performed using primers flanking differentially spliced regions, including one primer labeled with 6‐carboxyfluorescein (6‐FAM) for amplicon detection during fragment analysis. Cycle parameters included denaturation at 94°C for 2 min, followed by 24 cycles of 94°C for 15 s, 50°C for 30 s, and 70°C for 1 min, with a final elongation step of 72°C for 10 min. This was essentially the same regime described in (Stamm et al., 2012) but with fewer cycles to ensure reactions were stopped before exiting the logarithmic phase. PCR products were combined with size standards and separated on a 3730 Genetic Analyzer (Life Technologies). Amplicons were quantified using manufacturer‐provided software by calculating the area under each amplicon peak. The percentage of the variant containing the alternatively spliced region (%L, see above) was calculated by dividing the long form area by the total area for both long and short forms. Spreadsheets with data exported from the instrument, along with PSI calculations, are available in the project git repository (https://bitbucket.org/lorainelab/ricealtsplice) in a subfolder named “Experimental Testing.”

## 3 RESULTS

### 3.1 Most genes annotated as alternatively spliced favored one dominant form in rice

RNA‐Seq read alignments from our previously generated rice libraries were used to assess alternative splicing in four sample types: roots and shoots from seedlings treated with the cytokinin compound benzyladenine (BA) or with a mock, control treatment (Raines et al., 2016). Using the exon‐intron overlap method described previously (English et al., 2010), alternative splicing events within each gene were identified and annotated (Figure 1). Gene models that included an alternatively spliced region were designated the “L‐long” alternative form and models that lacked an alternatively spliced region were designated “S‐short”. For each alternative splicing event, the number of sequence alignments unambiguously supporting each alternative (L or S) was counted. These counts were then used to calculate percent‐spliced‐in (PSI), the percentage of read alignments supporting the longer (L) form.

In the combined data from all libraries from the rice data set there were 11,192 AS events. Of these, 77% had at least one read supporting each of the two splicing choices, and 19.8% had support for just one splicing choice. Only 2.8% of AS events has no reads supporting either form; these corresponded to genes with low or no expression in any of the sample types tested.

**Figure 2:**
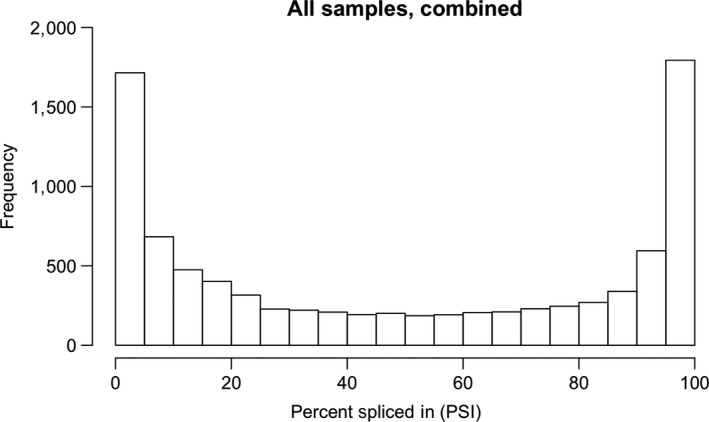
Distribution of percent‐spliced‐in (PSI) for annotated splicing events in rice where each choice was supported by at least one RNA‐Seq alignment. The *y* axis is the frequency of splicing events, and the *x* axis the PSI between 0 and 100%. PSI was calculated as 100*L/(S + L), where L and S were the number of reads that supported the splicing choice that included (L) or excluded (S) the differentially spliced region. Read alignment counts from all 12 libraries were combined to obtain a global view of alternative splicing occurrence in rice seedlings. The U‐shaped character of the distribution persisted whether lower or higher thresholds of informative reads were used

We further analyzed the 77% of AS events that had at least one read supporting each of the two splicing choices (L and S). Most genes annotated as alternatively spliced favored a single transcript, that is, 70% of the splicing events had a PSI <20% or >80% (Figure 2). Nevertheless, 29% of the splicing events (representing 1,861 genes) had a PSI between 20% and 80%, indicating that many genes produce multiple transcript variants (Figure 2).

### 3.2 Rice genes with abundant support for both alternative splicing choices perform many diverse functions

We used Gene Ontology term enrichment to determine if the subset of genes in rice for which alternatively spliced forms were unusually abundant exhibited enrichment with specific functions or processes, for example, circadian cycling, in which alternative splicing might play a prominent regulatory role. We asked if some Gene Ontology terms were significantly enriched with genes containing alternative splicing events with a PSI between 20% and 80%, corresponding to the central region of Figure 2. Interestingly, we found that these genes exhibited a diversity of gene functions, with no significant enrichment of functional categories. Thus, alternative splicing in which both transcripts are prevalent appears to affect genes with many functions in rice.

### 3.3 Many rice genes are differentially spliced between roots and shoots but cytokinin hormone application has minimal effect on splicing

In animals, differential splicing between cell or tissue types contributes to cellular differentiation, especially in the nervous system (Naftelberg, Schor, Ast, & Kornblihtt, 2015). Less is known about the role of differential splicing in regulating cellular differentiation and other processes in plants. Rice shoots and roots are profoundly different tissues, but our previous analysis of rice RNA‐Seq data found that many of the same genes were expressed in both tissues (Raines et al., 2016). This raises the question of how these two different tissues are able to carry out their specialized roles, and suggest the hypothesis that differential splicing could enable differential functions in genes expressed in both tissues, as proposed in Reddy et al. (2013).

Our previous analysis of the effects of cytokinin treatment on rice identified many thousands of genes that were differentially expressed in response to cytokinin (Raines et al., 2016). However, little is known about the role of alternative splicing during cytokinin response, except for one study in *Arabidopsis* that reported a shift in splicing of SR protein genes following cytokinin hormone treatment (Palusa et al., 2007). Therefore, we examined differential splicing in the rice RNA‐Seq data set comparing root and shoot tissue with or without cytokinin.

**Figure 3:**
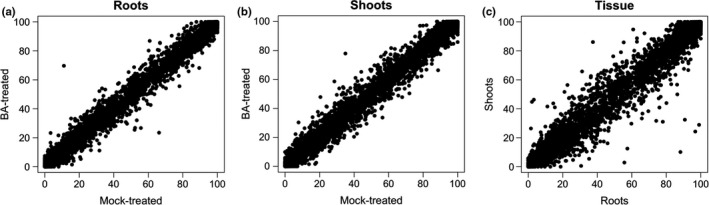
Scatter plots comparing percent‐spliced‐in (PSI) between sample types in rice for annotated splicing events. PSI was calculated from RNA‐Seq reads obtained from sequencing rice‐seedling shoots and roots grown hydroponically and subjected to a 2‐hr treatment with BA, a cytokinin analog, or a mock‐treatment (control). PSI is the average of three biological replicates. Only events with at least 15 informative read alignments in all six samples being compared were included. (a) BA‐treated rice roots (*y* axis) compared to mock roots (*x* axis). (b) BA‐treated rice shoots (*y* axis) compared to mock shoots (*x* axis). (c) Mock shoots (*y* axis) compared to mock roots (*x* axis)

First, we asked: When an alternatively spliced gene was expressed in two different sample types, was the relative abundance of splice variants the same or different? To address this, we examined the correlation of PSI between roots and shoots or between BA‐treated and mock‐treated samples (Figure 3). We found that PSI was similar between BA‐treated and untreated samples, as revealed by the tighter clustering of scatter plot points (Figure 3a,b). This indicated that genes that were alternatively spliced in BA‐treated samples were also alternatively spliced in the controls, and that the relative abundance of splice variants was similar. Thus, the cytokinin hormone treatment had minimal effect on splicing. By contrast, there were many genes where the relative abundance of splice variants was different between roots and shoots (Figure 3c). Consistent with Figure 3, statistical testing of PSI differences between sample types identified 90 genes, where PSI was significantly different between roots and shoots (FDR ≤ 0.1), but only four and two genes where PSI was different between BA‐treated samples and controls in roots and shoots, respectively (Table S1). Thus, we observed limited but non‐trivial levels of differential alternative splicing between roots and shoots but minimal differential alternative splicing between control and BA‐treated samples.

### 3.4 Comparison of *Arabidopsis* differential splicing shows similar patterns to rice

To determine whether the observed patterns of differential splicing are similar in other plants, we analyzed splicing in *Arabidopsis* roots and shoots that had also been treated with the cytokinin compound benzyladenine in our previous study (Zubo et al., 2017). Due to the *Arabidopsis* libraries not being sequenced to the same depth as the rice libraries, many more splicing events had little or no support. Using the same FDR threshold as with the rice data set (FDR ≤ 0.1), we identified few differentially spliced regions between shoots and roots (3) and none in the control to treatment comparisons (Table S2). However, PSI was distributed similarly to rice in that most alternatively spliced genes expressed one major form (Figure S1a). In addition, scatter plots showing average PSI in treated versus untreated samples showed a much tighter clustering of points as compared to scatter plots comparing roots and shoots (Figure S1b–d). Statistical testing of PSI differences confirmed the cytokinin hormone treatment had minimal effect on splicing in *Arabidopsis*. Thus, the general pattern of more differential splicing between tissue types as compared to treatment with exogenous cytokinin appears conserved between rice and *Arabidopsis*.

### 3.5 Alternative splicing remodeled protein‐coding sequence more often than disrupting it in rice

Alternative splicing can occur anywhere in a gene, including UTR and protein‐coding regions. Because three bases encode one amino acid, the lengths of spliced coding regions in a transcript are multiples of three. Thus, when alternatively spliced regions occur in coding regions and are not multiples of three, then inclusion of these regions in transcripts is likely to introduce a frame shift, resulting in a premature stop codon and a truncated protein product. As shown in Table 1, there was an enrichment of alternatively spliced regions in rice that were evenly divisible by three in coding regions versus non‐coding in all subsets of the data. These subsets included all annotated alternatively spliced regions, regions where the minor form was prevalent (the region between 20% and 80% PSI in Figure 2), and differentially spliced regions. Thus, alternative splicing within the coding regions of genes was biased against introducing frame shifts and promoted protein remodeling rather than truncation.

**Table 1:** Counts of alternative splicing choices in rice where the length in nucleotides of the splicing event is evenly divisible by three or with remainder of 1 or 2. A remainder of 1 or 2 leads to a shift in frame in translation. A *p*‐value < 0.05 is considered significant

Alternative splicing		Divisible by 3	Remainder of 1	Remainder of 2	*p*‐Value^a^
Location	Event				
Coding region	Annotated as alternatively spliced	3,248	2,466	2,411	3e‐36
UTR	Annotated as alternatively spliced	1,152	1,127	1,113	1
Coding region	Minor form is expressed	173	149	153	8e‐6
UTR	Minor form is expressed	34	20	13	0.03
Coding region	Differentially spliced	33	24	18	0.03
UTR	Differentially spliced	6	5	11	0.79

a *p*‐Value obtained from binomial test of the null hypothesis that the true probability of a differentially spliced region having a length divisible by three is 1 in 3 and an alternative hypothesis that the probability is greater than 1 in 3.

Most differential splicing between roots and shoots (67%) occurred within protein‐coding regions (Table S1), suggesting that differential splicing is likely to affect gene function at the level of the protein product. In every instance of differential splicing, major and minor forms were both detected, with differential splicing observed as a change in the relative abundance of the two forms.

To further understand the effects of splicing on protein‐coding sequences, we visualized differentially spliced regions together with RNA‐Seq alignments, coverage graphs, and inferred junctions using genome browsers. Two genome browsers were used to visualize the data—Integrated Genome Browser (Freese et al., 2016) and ProtAnnot (Mall et al., 2016). Integrated Genome Browser (IGB) was used to examine RNA‐Seq read alignments and compare alignments to the annotated gene structures. ProtAnnot, an IGB App, was used to search the InterPro database of conserved protein domains to find out how (or if) splicing inferred from RNA‐Seq data was likely to affect gene function through remodeling of protein domains as detected by the InterProScan Web service (Finn et al., 2017).

**Figure 4:**
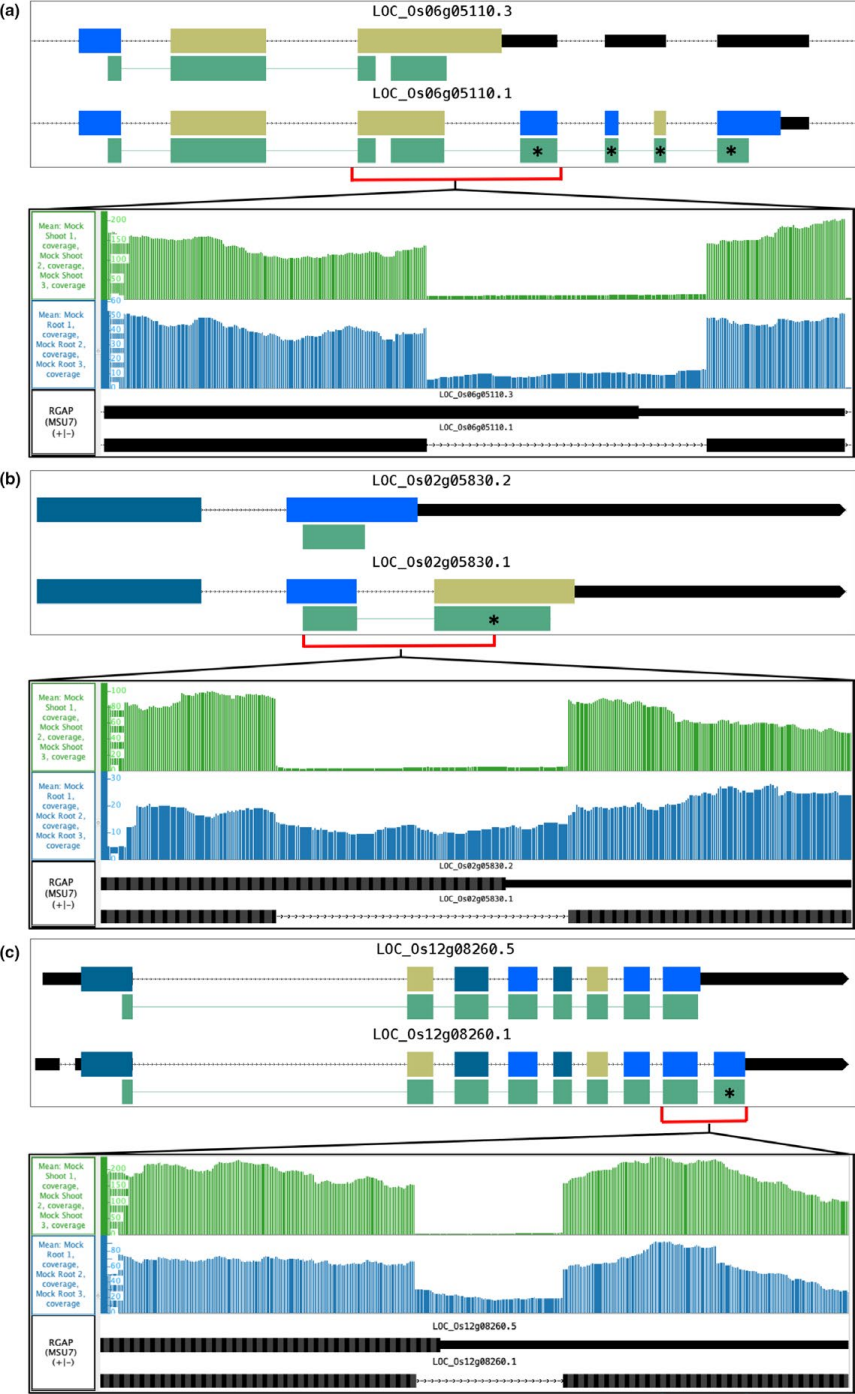
ProtAnnot and IGB images showing differences in splicing between rice shoot and root. ProtAnnot (upper panels) shows coding region exons color‐coded by frame, with regions matching InterPro profiles indicated by green, linked rectangles. Asterisk characters highlight differences in profile matches. Integrated Genome Browser (lower panels) shows a zoomed‐in view of RNA‐Seq coverage graphs from rice root (blue) and shoot (green). *Y* axis is the number of RNA‐Seq aligned sequences with MSU7 gene models in black below. (a) LOC_Os06g05110 isoforms .3 and .1 encoding superoxide dismutase, matching InterPro profile IPR019831 and IPR019832. (b) LOC_Os02g05830 isoforms .2 and .1 encoding RuBisCO small subunit, matching InterPro profile IPR000894. (c) LOC_Os12g08260 isoforms .5 and .1 encoding dehydrogenase E1 component, matching InterPro profile PTHR11516

Of the 105 differentially spliced regions between rice roots and shoots, 71 overlapped protein‐coding sequence regions, suggesting that in these cases, splicing affected protein function. All but one (70/71) of the differentially spliced regions embedded in coding regions overlapped a predicted functional domain (e.g., a predicted transmembrane helix) or a region found by protein classification systems (e.g., Pfam (Finn et al., 2016) or PANTHER (Thomas et al., 2003)) to be conserved among members of the same protein family (Table S1 and Figure 4).

Figure 4a shows LOC_Os06g05110, encoding a putative chloroplast superoxide dismutase enzyme. Superoxide dismutase enzymes protect the plant from reactive oxygen species by catalyzing toxic superoxide radicals to molecular oxygen (Alscher, Erturk, & Heath, 2002). Transcript LOC_Os06g05110.3 contained a retained intron, which caused a frame shift, a premature stop codon, and a truncated match to a manganese/iron superoxide dismutase C‐terminal profile (Figure 4a). The lower panel of 4A shows RNA‐Seq coverage graphs for shoots and root samples. Roots had a higher relative proportion of read support for the retained intron (long) form (38.5% in root, 13.6% in shoot) (Table 2). Average expression in roots was lower than in shoots, 12.5 versus 39.7 Reads Per Kilobase per Million mapped reads (RPKM) (Table 2). Thus, roots had lower overall expression of LOC_Os06g05110 and an increased relative proportion of the retained intron transcript with a shortened truncated match to a manganese/iron superoxide dismutase profile.

**Table 2:** Differential splicing of 10 genes in rice. Genes were first detected using RNA‐Seq and re‐tested using capillary gel electrophoresis (CGE). A *p*‐value < 0.05 is considered significant

Gene	AS type	Avg. RPKM expression		RNA‐seq PSI (%L)		CGE PSI (%L)		*p*‐Value^a^
		Root	Shoot	Root	Shoot	Root	Shoot	
LOC_Os01g25484, ferrodoxin nitrite reductase	RI	300	131	74.2	31.7	29.9	13	5e‐05
LOC_Os01g35580, unknown	AS	55.7	34.9	44.0	66.3	49.9	66.9	2.47e‐04
LOC_Os01g45274, carbonic anhydrase	ES	171	1,380	96.8	24.3	97.4	15.4	3e‐09
LOC_Os01g51290, protein kinase	RI	49.6	49.9	88.4	95.1	13.3	17.2	0.03466
LOC_Os03g05390, citrate transporter	RI	219	174	86.0	96.3	86	95.5	1.2e‐04
LOC_Os12g08260, dehydrogenase E1	RI	8.03	30.3	55.7	2.9	4.1	0.87	3e‐05
LOC_Os01g61670, ureidoglycolate hydrolase	DS	59	37.8	78.0	31.0	59	37.8	1.6e‐09
LOC_Os05g48040, MATE efflux family protein	DS	7.05	6.72	88.1	100.0	89.4	88.27	0.632
LOC_Os02g05830, ribulose bisphosphate carboxylase	RI	4.79	12.3	88.2	10.1	23.9	3.06	8e‐04
LOC_Os06g05110, superoxide dismutase	RI	12.5	39.7	38.5	13.6	22.7	6.5	1.3e‐07

a *p*‐Value obtained from comparing roots and shoots PSI from CGE.

Figure 4b shows LOC_Os02g05830, which codes for ribulose bisphosphate carboxylase (RuBisCO) small chain chloroplast precursor. Retained intron event in transcript LOC_Os02g05830.2 caused a frame shift, a premature stop codon, and alternative carboxy terminal regions. This resulted in a shorter match to a RuBisCO small subunit profile from the Interpro database, as shown in the upper panel of 4B. The bottom panel of 4B shows that roots had an increased proportion of reads supporting the retained intron (long) form (88.2% in root, 10.1% in shoot) as well as lower overall expression compared to shoots, 4.8 versus 12.3 RPKM (Table 2).

Rice contains five RuBisCO small subunit genes with varying expression levels, but only LOC_Os02g05830 was differentially alternatively spliced between roots and shoots. The relatively low levels of expression of LOC_Os02g05830 contrasted with the expression of the paralogous RuBisCO small subunit gene LOC_Os12g19470 (80.2 and 942 RPKM in roots and shoots). LOC_Os12g19470 also contained an alternative spliced, retained intron form that introduced a frameshift and premature stop codon. However, we did not find statistically significant differential splicing between roots and shoots for LOC_Os12g19470. Its proportion of long to short form splicing was stable across tissues and expression levels. Understanding how these splicing ratios were maintained is beyond the scope of this study, but the existence of differentially spliced paralogs may provide an opportunity to identify sequence features that stabilize splicing.

Figure 4c shows LOC_Os12g08260, encoding dehydrogenase E1 component domain containing protein. In InterPro, the dehydrogenase E1 component domain is annotated as belonging to many dehydrogenases of diverse biological function. Thus, there is no known function for LOC_Os12g08260 apart from its putative enzymatic activity. The top panel of Figure 4c shows that transcript variant LOC_Os12g08260.5 contained a retained intron event that introduced a frame shift. The frame shift changed the carboxy terminus of the protein and disrupted a profile match to the dehydrogenase E1 component domain. The bottom panel of Figure 4c shows that the root sample had an increased proportion of reads supporting the retained intron (long) form (55.7% in root, 2.9% in shoot), and gene expression was lower overall in roots (8.0 vs. 30.3 RPKM) (Table 2).

### 3.6 RT‐PCR with capillary gel electrophoresis confirmed differential splicing between rice roots and shoots for 9 of 10 genes tested

We used a method based on capillary gel electrophoresis of fluorescently tagged PCR products to assay splicing of 10 genes detected as differentially spliced between rice roots and shoots (Stamm et al., 2012). New rice seedlings were grown under a close‐to‐identical replication of the RNA‐Seq experiment. Primers were designed to amplify differentially spliced regions, including one primer that was conjugated to a fluorescent tag. Following PCR amplification of cDNA prepared from the new rice samples, products were resolved on a capillary‐based sequencer and PSI calculated (Table 2). In 9 out of 10 genes, differential alternative splicing was confirmed. In the one case where differential alternative splicing was not confirmed, there were very few RNA‐Seq read alignments covering the differentially spliced region, suggesting this was likely a false positive result. The FDR cutoff used to detect differential splicing in the RNA‐Seq data was 0.1, corresponding to 1 in 10 false discoveries, in line with results from the microcapillary‐based analysis.

## 4 DISCUSSION

This study profiled alternative splicing using a high coverage RNA‐Seq data from 10‐day old, hydroponically grown rice seedlings treated with a cytokinin hormone. A less deeply sequenced data set from cytokinin‐treated *Arabidopsis* seedlings provided a comparison between monocot versus eudicot. We found that cytokinin treatment induced very few splicing changes between treated and untreated controls for both rice and *Arabidopsis*. However, there were many differences in splicing between untreated rice roots and shoots, and most of these changed the protein‐coding region of genes.

Palusa et al. (2007) found that BA‐treatment of *Arabidopsis* seedlings triggered splicing changes in multiple SR genes, encoding RNA‐binding proteins whose counterparts in metazoans regulated alternative splice site choice. Their study used PCR amplification of cDNA followed by agarose gel electrophoresis to detect changes in splicing and focused on SR protein genes only. Thus, we expected to observe changes in SR gene splicing due to the cytokinin treatment, leading to changes in splicing for many downstream genes. However, close examination of SR splicing genes within our RNA‐Seq datasets revealed no statistically significant differential splicing due to BA‐treatment. It is possible that the differences in methodology used to measure splicing changes between the two studies (RNA‐Seq vs. visualization of PCR amplification of cDNA) could account for the differences in observations.

One possible explanation for why the cytokinin treatment had minimal effect on splicing was that the treatment itself was ineffective. However, differential expression analysis showed that many genes were up‐ or downregulated by the treatment in the two data sets tested—rice and *Arabidopsis* (Raines et al., 2016; Zubo et al., 2017). Fewer genes were detected as differentially expressed in the *Arabidopsis* data set, most likely reflecting higher variability between biological replicates combined with lower sequencing depth as compared to the rice data set. Nevertheless, known cytokinin response genes were differentially regulated in both data sets, showing the cytokinin treatment penetrated plant cells and induced stereotypical cytokinin signaling without also triggering changes in splicing.

The relative lack of differential splicing between cytokinin‐treated and mock‐treated samples suggests that cytokinin signaling does not employ alternative splicing as a regulatory mechanism to the same degree as with other plant hormones, notably abscisic acid (ABA). ABA plays a role in perception and response to stresses, especially desiccation stress (Maia, Dekkers, Dolle, Ligterink, & Hilhorst, 2014). ABA also plays a role in regulating splicing of SR45, an SR‐like protein, and SR45 plays a role in regulating downstream splicing of multiple genes (Cruz, Carvalho, Richardson, & Duque, 2014). Thus, there is a clear connection between stress signaling and the plant stress hormone ABA.

By contrast, cytokinin signaling involves transfer of phosphate groups between successive elements of a phosphorelay signaling pathway culminating in phosophorylation‐dependent activation of Myb‐type transcription factor proteins called type B ARRs. Cytokinin treatment has no or little effect on transcription of type B ARRs, the key regulators of cytokinin signaling (Argyros et al., 2008; Kieber & Schaller, 2018). In addition, type B transcriptional regulators are not highly alternatively spliced. By contrast, a closely related family of similar genes encoding so‐called “pseudo‐response regulators” have similar sequence to type B ARRs and are highly alternatively spliced (James et al., 2012). These genes are involved in regulating the circadian clock and have nothing to do with cytokinin signaling.

Using the same methods and data set, we identified a relatively large number of genes in rice (90) that were differentially spliced genes between shoots and roots, and we validated 9 of 10 using fragment analysis of independently produced rice samples (Tables S1 and 2). This observation of differential splicing between roots and shoots is important for two key reasons. First, it shows that our data analysis methods can identify differential alternative splicing in a data set. In other words, the roots versus shoots comparison provided a positive control for differential splicing detection. Second, the detection of differential splicing between roots and shoots illuminates the function of alternative splicing in plant cells. Our data supports the growing body of evidence that alternative splicing is cell, tissue, and stage specific in plants (Gupta et al., 2015; Sun et al., 2018; Vitulo et al., 2014), including in roots (Li et al., 2016).

Interestingly, in the three examples highlighted in Figure 4, the proportion of retained intron form was anti‐correlated with transcript levels overall. In all three cases, the retained intron led to a premature stop codon and truncation of the predicted domain. In general, superoxide dismutase and RuBisCO are important in photosynthetic tissue, and LOC_Os06g05110 and LOC_Os02g05830 had higher expression in shoots than roots (Alscher et al., 2002; Andersson & Backlund, 2008). Although we cannot say to what extent alternative splicing affected the protein function, we presume that the truncated domains would have a functional difference. As expression is already decreased in roots compared to shoots for LOC_Os06g05110 and LOC_Os02g05830, the increased proportion of retained intron in roots (and thus truncated domain) may act to further downregulate the number of fully functional proteins. It is likely that alternative splicing plays a role in fine‐tuning gene function to meet the needs of different plant tissues or cell types where a gene is expressed.

We also examined the prevalence of alternative splicing, independent from differential splicing. That is, we used RNA‐Seq read alignments to assess how often annotated alternative splice sites were used in our RNA‐Set data sets. For most genes annotated as alternatively spliced, the minor form frequency was typically low, accounting for <20% of sequence read alignments across the differentially spliced region. Genes where minor form frequency exceeded 20% exhibited a diversity of functions. Thus, many diverse processes in rice involved alternatively spliced genes in which splice variants were expressed at levels likely to affect gene function in different ways.

A major limitation of this study was that we limited our analysis to annotated splice forms and did not attempt to form new transcript models based on the RNA‐Seq data. This was done mainly because the libraries used were not strand‐specific and attempts to assemble transcripts using transcript assembly tools led to incorrect fusion of neighboring genes and other artifacts (not shown). Future studies will therefore benefit greatly from using better library preparation protocols to simplify and streamline data analysis. Nonetheless, this analysis provides new insight into the role of alternative splicing in plant tissues and hormone response.

In conclusion, by analyzing the number of reads that supported different splice variants, we identified examples of differential splicing with confirmation by RT‐PCR with capillary gel electrophoresis. There were 90 genes differentially spliced between rice root and shoot tissues, but only four between cytokinin‐treated and non‐treated samples. For most differential splicing events, the protein‐coding regions were affected, strongly suggesting that differential splicing is playing a role in modulating gene function between roots and shoots.

## CONFLICT OF INTEREST

The authors declare no conflict of interest associated with the work described in this manuscript.

## AUTHOR CONTRIBUTIONS

AEL designed the research. ARE and ICB performed the experiments. NHF, ARE, ICB, JD, and AEL analyzed the data. NHF, ARE, and AEL drafted the manuscript with contributions from ICB and JD.
