# Supplementary figures and images for: Many rice genes are differentially spliced between roots and shoots but cytokinin has minimal effect on splicing
Source: Plant Direct. 2019 May 17;3(5):e00136. doi: 10.1002/pld3.136 (PMC6589529; doi:10.1002/pld3.136)

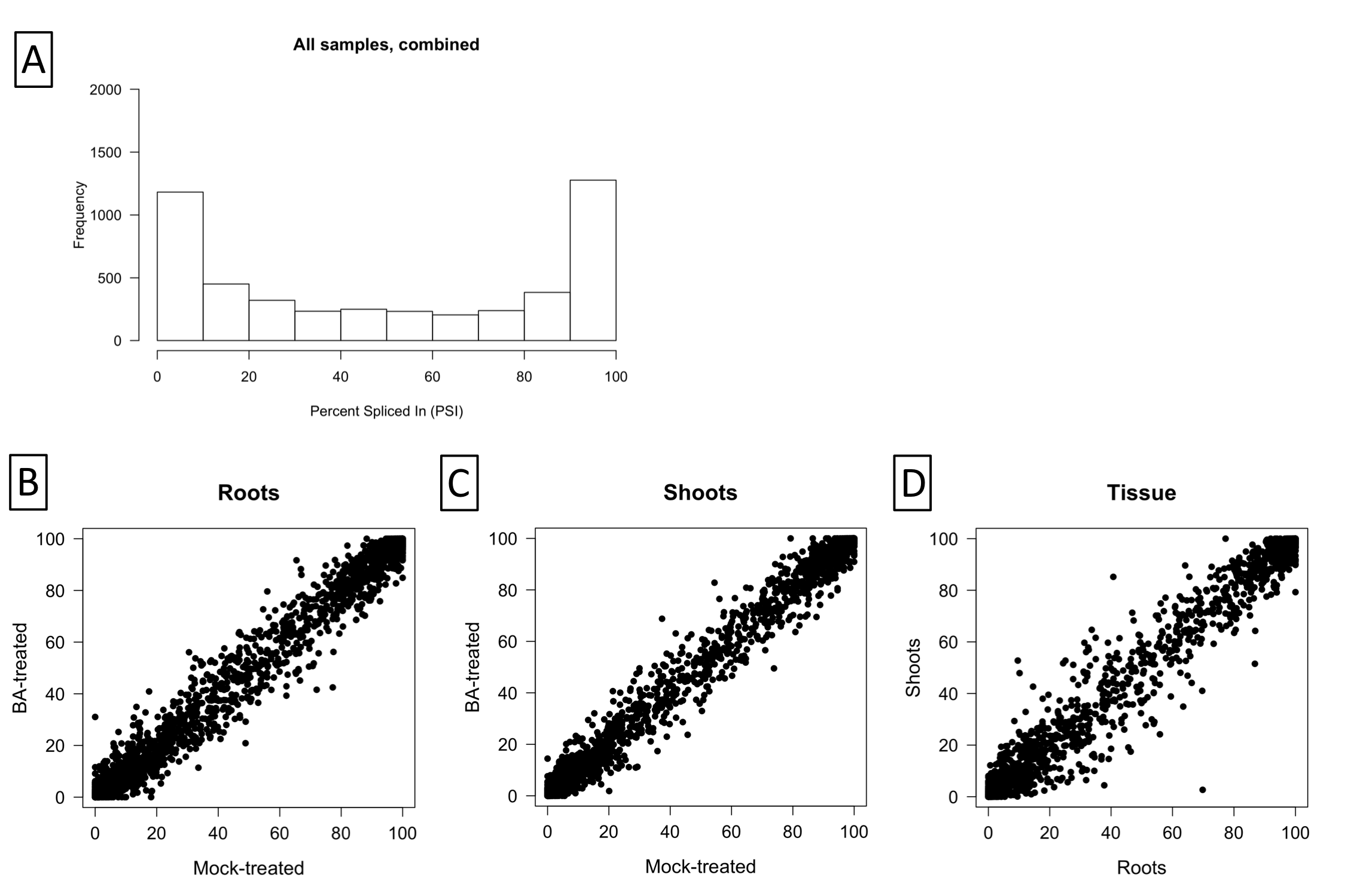

Supplement: Supplementary file 1 [file PLD3-3-e00136-s001.png]
